# Exploring nurse and nursing student experience of using an artist-produced photobook to learn about dementia

**DOI:** 10.1186/s12912-022-00991-2

**Published:** 2022-08-25

**Authors:** Savannah Dodd, Gillian Carter, Andrena Christie, Gary Mitchell

**Affiliations:** 1 School of History, Anthropology, Philosophy, and Politics, Queen’s University Belfast, 25 University Square, County Antrim, Belfast, BT7 1NN Northern Ireland UK; 2 School of Nursing and Midwifery, Medical Biology Centre, Queen’s University Belfast, 97 Lisburn Road, County Antrim, Belfast, BT9 7BL Northern Ireland UK

**Keywords:** Nursing education, Dementia, Dementia education, Pedagogy, Photography, Photobook, Arts-based research, Arts-based pedagogy, Visual learning, Person-centred care

## Abstract

**Background:**

Improving understanding about dementia in nursing is a priority area for educators and policymakers. This is due to poor professional understanding about dementia and suboptimal healthcare practice. While many educational interventions exist, there has been a paucity of research which has considered the use of artist-produced photobooks to improve knowledge and understanding about dementia. The aim of this study is to understand the impact of an artist-produced photobook on nurses’ attitudes and beliefs about dementia.

**Results:**

Following a thematic analysis of four focus group interviews with 22 nurses and nursing students from Northern Ireland, three themes emerged. Theme one was about how the artist-produced photobook helped participants to humanise the person living with dementia. Theme two related to how the artist-produced photobook supported participants to actively construct their own meanings about dementia based on their previous professional and personal experiences. Theme three explored how an artist-produced photobook could be successfully used to complement existing dementia education in the future.

**Conclusions:**

Using an artist-produced photobook was an innovative way to learn about dementia for nurses and nursing students. The photobook functioned as a tool underpinned by arts-based pedagogy (ABP), supporting nurses to understand the person behind the dementia disease. As such, an artist-produced photobook has the potential to be a useful complementary resource for supporting professional education about dementia.

**Supplementary Information:**

The online version contains supplementary material available at 10.1186/s12912-022-00991-2.

## Background

Dementia affects more than 50 million people globally with an estimated 10 million new cases annually [1]. This disease may result from approximately 200 different conditions, but the most common are Alzheimer’s disease, vascular dementia, Lewy-body dementia, and frontotemporal dementia [2]. While the clinical manifestations of dementia are always unique to the person, the disease is typically characterised by short-term memory loss, communication difficulties, progressive functional decline, personality changes, and distress [3–5]. Despite increasing prevalence, healthcare professional understanding about dementia remains low. A recent international survey of more than 14,000 healthcare professionals indicated that 62% believed that dementia was an inevitable and normal part of ageing [6]. Poor professional understanding about dementia can lead to suboptimal patient experience, complex behaviours, and adverse clinical outcomes [7].

There has been a plethora of empirical research and critical discussion which has highlighted how healthcare professionals can inadvertently disempower people with dementia due to this misunderstanding by, for example, focusing on disability not capability, using stigmatising language, excluding the person from treatment decision-making, or curtailing the person’s independence [8–13]. It is therefore important that all healthcare professionals, formal caregivers, and healthcare students receive education about dementia which they can readily apply to their practice [14]. Over the past decade, there have been several innovative approaches to providing education to this population about dementia [15–20]. These approaches to nursing education have focused on a wide range of aspects including dementia awareness, communication, diagnosis, person-centred care, promotion of enabling environments, palliative care, family partnerships and evidence-based practice [15–20].

Nurses, and nursing students, occupy an important role for people with dementia throughout the course of their disease [21]. As the largest single group of healthcare professionals globally, they have an important role in primary, secondary, and tertiary care, and are likely to support the person from pre-diagnosis to end-of-life care [22–24]. Whilst there are many types of dementia education, this study sought to explore the experiences of nurses and nursing students that received dementia education using an artist-produced photobook which depicts the journey of a person living with dementia. The objective of this study was to understand the impact of the photobook on nurses’ attitudes and beliefs about dementia. To our knowledge, this is the first time an educational intervention of this type has been explored with nursing students and registered nurses.

The Visual Auditory Read/Write Kinaesthetic Model (VARK) model, developed by Fleming and Baume, suggests that people have different educational preferences that correspond to different learning styles. For example, visual learners prefer to learn by watching, auditory learners prefer to learn by listening and kinaesthetic learners prefer to learn by doing. Embedding a visual learning tool within a traditional curriculum can help to cater to all students within a cohort who might have different learning styles [25].

The use of visual learning tools in nursing education constitutes a form of arts-based pedagogy (ABP). According to Rieger et al., ABP is a method of teaching in which a student learns about a topic through the process of creating or responding to a piece of artwork [26]. The use of ABP in nursing education has been linked to improving “nursing students’ knowledge acquisition, level of empathy, attitude toward others, emotional states, level of reflective practice, learning behaviors and aspects of cognitive/ethical maturity” [26]. Moreover, by putting value on “both personal and aesthetic ways of knowing,” ABP can inspire nurses to develop more meaningful relationships with the people in their care [27]. Photography is one artform that has been used in ABP.

Photography has been used in a variety of ways to improve research about dementia and dementia care in recent years. Ward explains that there are many opportunities for using creative methods in participation with people who have dementia, and that such interventions can have positive impacts on an individual’s wellbeing [28]. Furthermore: “The use of photography and storytelling have also been used successfully to hear the voice of the person with dementia, to help them become active members of the research process and have been found to be an empowering process, helping to develop a sense of identity through participation” [28]. Thus, some studies have used photovoice and photo elicitation with people with dementia and their carers as a research tool to better understand the lived experience of caring for people with dementia [29–31] and of life after a dementia diagnosis [28–33]. Other studies have tested whether viewing artist-produced photographs can improve the mood, well-being, and social interactions of people with dementia [34–38]. However, few projects have used photography as a tool for nursing education about dementia [39], and none that we could find have previously employed a photobook in an educational intervention.

An artist-produced photobook is different from the widely used life-history photobooks. Life-history books are a tool for reminiscence for the individual with dementia, or for dementia healthcare professionals to better understand details about the practical preferences and real-life experiences of the individual in care [40]. Whereas individualised life-history photobooks are created for each person with dementia, this artist-produced photobook sought to depict one person’s experiences with a view to eliciting empathy and understanding about the broader experience of dementia. The rationale for this approach is supported by Tippin and Maranzan who assert that individuals who learn about the lived experience of a single person within a marginalised group, in this case a person living with dementia, are likely to develop empathetic feelings and that these feelings often generalise toward the larger group [41]. Therefore, the aim of this study was to explore the perceptions of nurses and nursing students’ who used the artist-produced photobook as a means of understanding the impact of it on nurses’ attitudes and beliefs about dementia.

## Methods

### Intervention

The development of the photobook was not part of this research study. The photobook was independently developed by the artist (SD) prior to partnership on this research intervention.

**Fig. 1:**
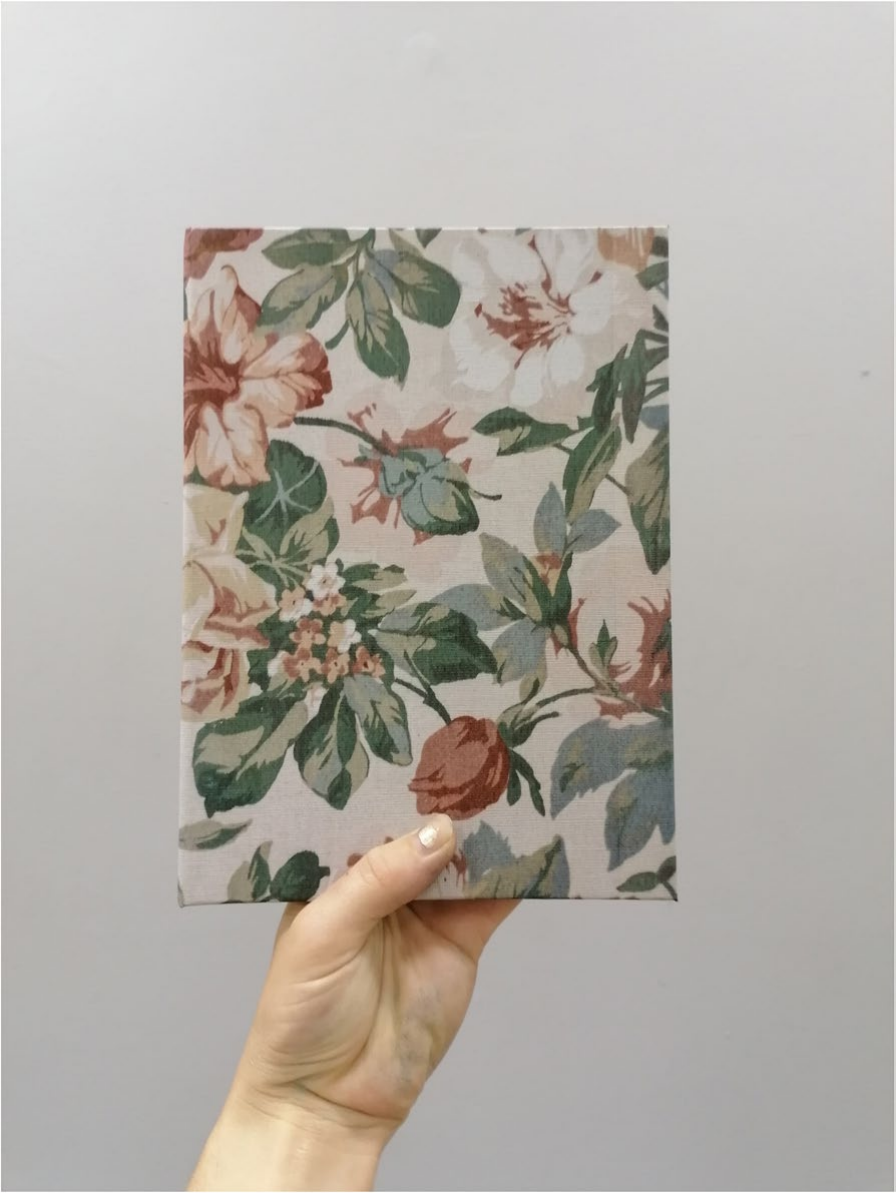
Cover of photobook

**Fig. 2:**
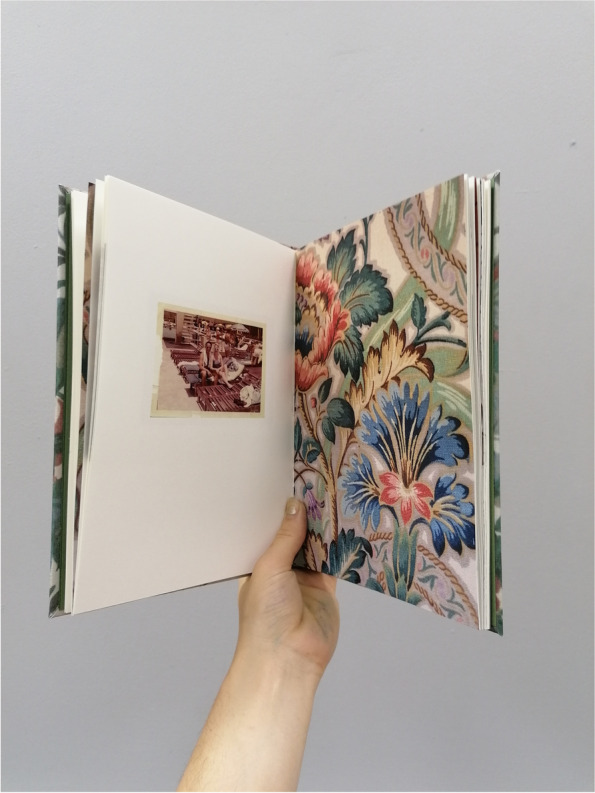
Inside of photobook

In October 2018, anthropologist and photographer (SD) began photographing her grandad who was in the later stages of dementia and was transitioning from his home of nearly 30 years to a memory care facility. Over the course of ten months, he was photographed using his own analogue Single-Lens Reflex (SLR) camera from the 1970’s. This was a deliberate artistic choice, because the experience of using film photography, compared to newer digital versions, has parallels with the unpredictability of memory, especially when impaired by dementia diseases [3–5]. In addition to making new photographs with the analogue SLR, digital photographs were taken of furnishing textures and wallpaper patterns in his home. As part of this process, the family’s photographic archive was reviewed, along with a leather-bound journal of handwritten entries that he wrote over the course of approximately eight years while his memory declined. In August 2019, these elements (film photographs of him, digital photographs of his home, archival family photographs, and excerpts of his handwritten journal entries) were compiled to create a unique photobook titled *Thanks, Gd* (Figs. 1 and 2).

**Fig. 3:**
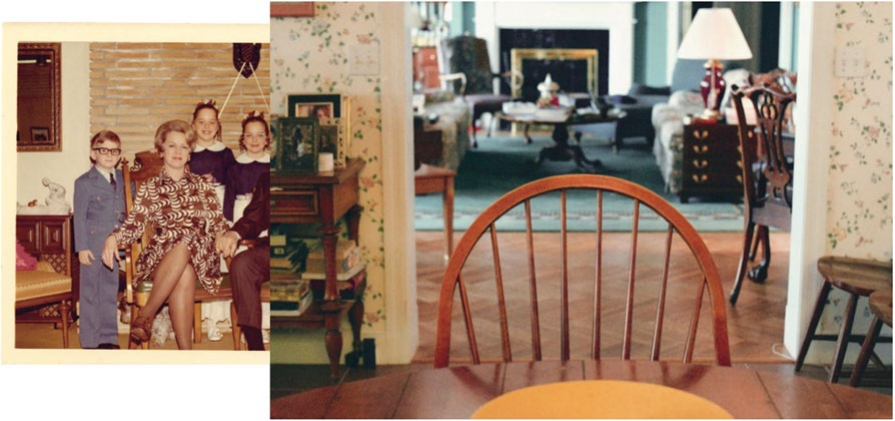
Page spread from inside the photobook showing pairing of archival and contemporary photographs

**Fig. 4:**
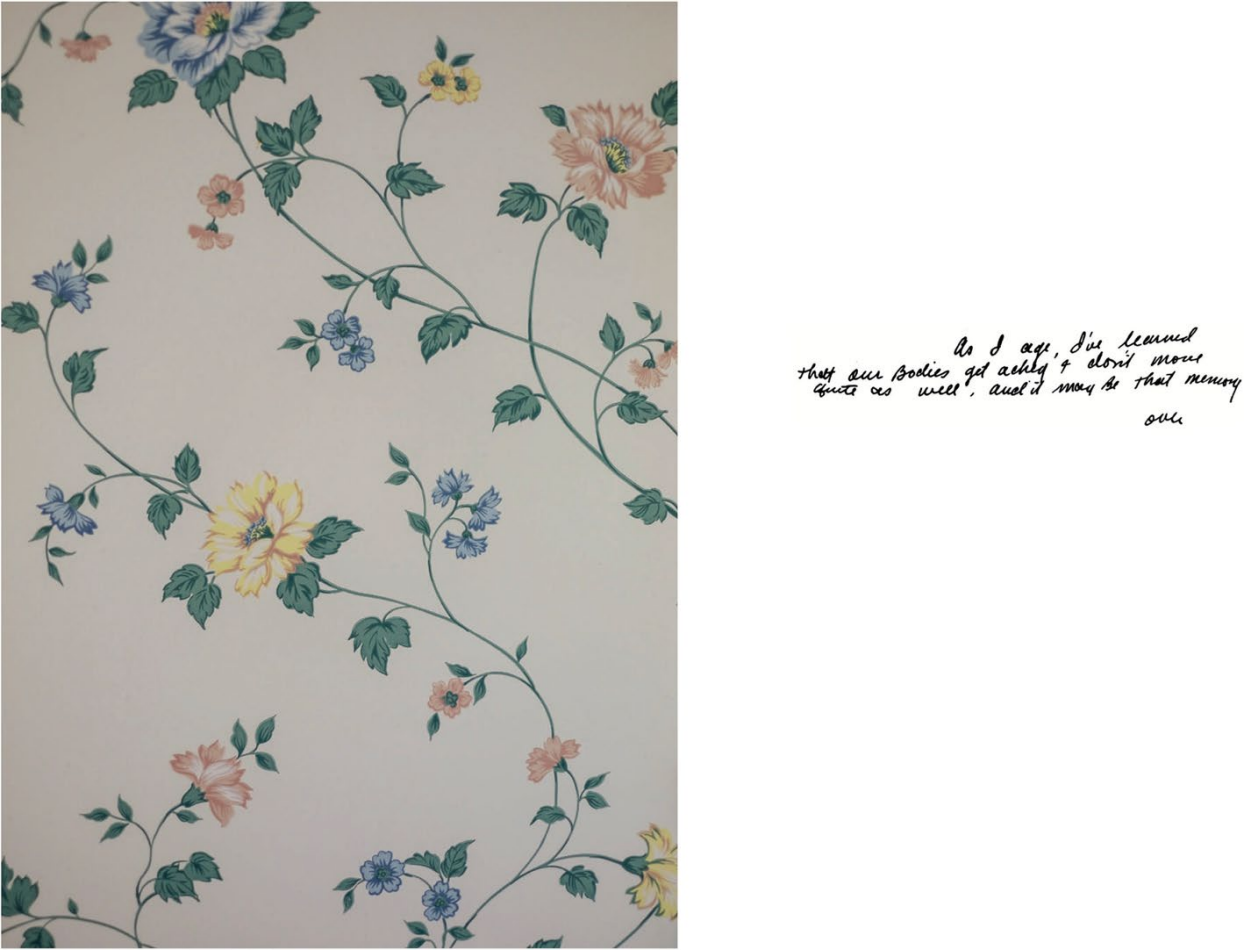
Page spread from inside the photobook showing a photograph of a wallpaper pattern and a handwritten note

**Fig. 5:**
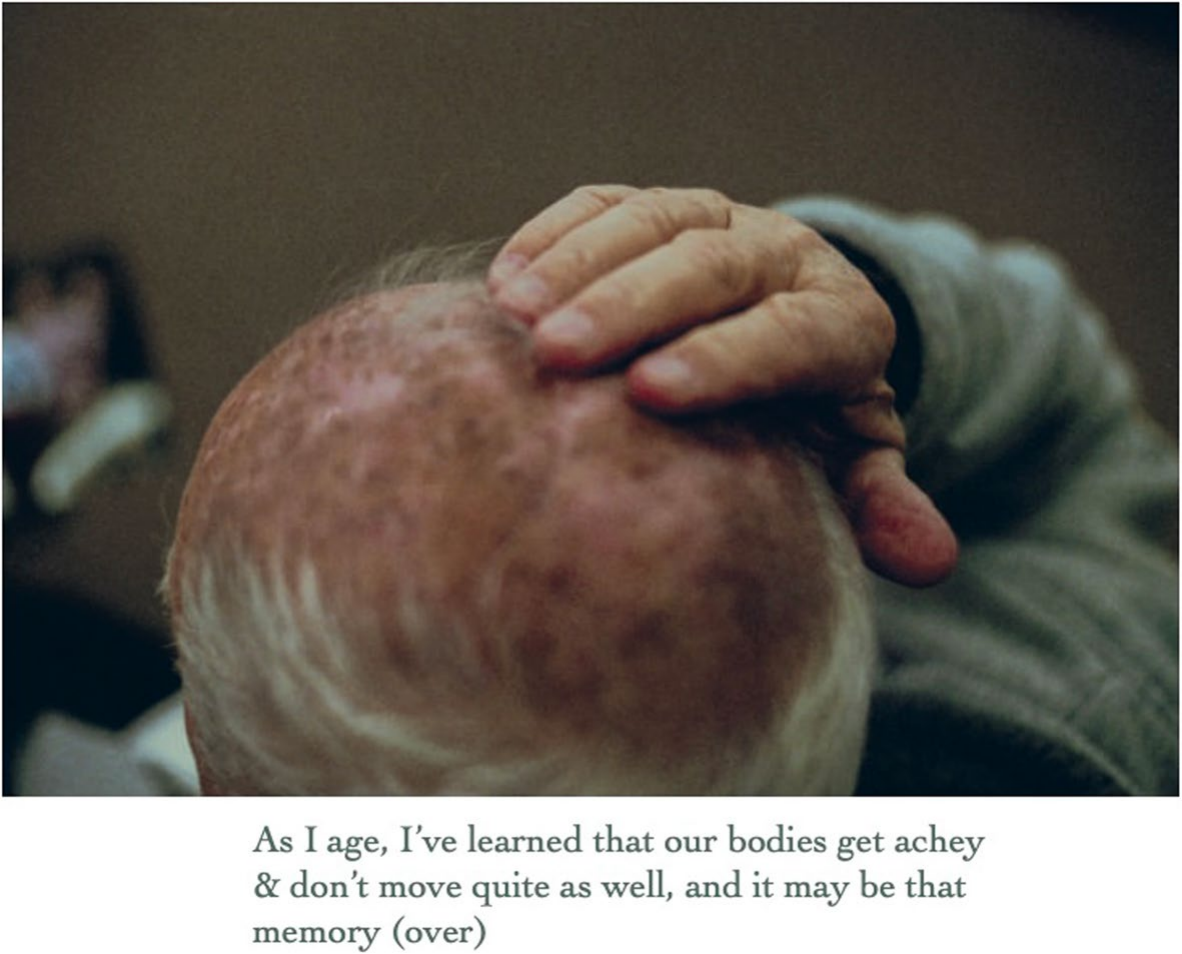
A montage of materials from a page spread showing a transcription of the handwritten note and a photograph of grandad

The photographs that appear inside the photobook are not presented in chronological order and are not labelled. In addition, archival photographs were paired with photographs from different decades (Fig. 3). This approach was intentionally used to distort time and create confusion, thereby promoting parallels with dementia [2–5]. To aid the reader, a postcard containing captions for all the archival photographs was placed about two-thirds of the way through the photobook. This placement late in the photobook encouraged the reader to go back and work through the photobook with the aid. Writings by the artist’s grandad were scanned to be included in handwritten form (Fig. 4). Typed transcriptions of these excerpts appeared on the backs of those pages (Fig. 5). At the end of the artist-produced photobook, there is an essay written by the artists’ grandmother to provide context on the role of the informal caregiver.

In this study, the hardbound photobook was printed using high-quality materials. Drawing from sensory anthropology, there is a strong argument for the importance of the materiality of the photobook as an object [42]. A high-quality, hardcover photobook was deemed as more likely to evoke a stronger emotional response from the viewer than a digital edition or a cheaper, disposable magazine.

Artwork was a key methodological tool for this study. In this way, this project could be considered a form of art-based research (ABR) [43]. ABR is highly compatible with constructivist pedagogy, “based on the co-construction of knowledge as a creative process” [44]. Therefore, the underpinning pedagogical approach to using the artist-produced photobook in nursing education was constructivist theory, whereby healthcare professional learners would actively construct their own knowledge and meanings about dementia through viewing the photobook. Constructivist theory is a popular pedagogical approach in higher education which has led to positive learning outcomes [45]. This pedagogical model is student-centred; students’ pre-existing knowledge, background experiences, and personal reflections are valued as important elements of the learning process.

### Design

An interpretive qualitative design was used to explore undergraduate and postgraduate nursing students’ perspectives of the artist-produced photobook. This design was chosen on the belief that participants would construct their own unique meanings of the photobook based on their previous professional and personal experiences of dementia [46]. This approach to education was based on an asynchronous methodology, with nurses and nursing students reviewing the artist-produced photobook in their own time outside of a formal classroom setting [47].

Data were collected using focus groups and this enabled nurses and nursing students to describe their own experiences of using the photobook and to also comment on the experiences of their peers [48].

### Ethics

The study obtained received ethical approval by Queen’s University Faculty Ethics Research Committee in September 2020 (reference MHLS 20_113). All participants received a written information sheet and completed an online informed consent form prior to their participation in the study. Verbal informed consent rechecked prior to commencement of all focus groups.

Informed consent to use written and visual materials about the artist’s (SD) grandad in the photobook was given by his wife and children. His wife, SD’s grandmother, was further consulted on the use of the photobook in this study prior to data collection [49]. All family members pictured in included images have given their informed consent for the publication of potentially identifying information/images in an online open-access publication.

### Recruitment

Eligible participants included students who were enrolled in either the Professional Nursing (BSc) undergraduate programme or continuous professional development postgraduate modules which focused on dementia at Queen’s University Belfast in Northern Ireland. These participants were identified by purposive sampling.

All eligible participants (*n* = 340) received an email about the opportunity to participate in this study and an information sheet by a gatekeeper who was not part of the study. Interested participants were invited to contact a member of the project team for further information about signing consent and participating in the study. Recruitment to this study concluded after 24 people agreed to participate and had signed consent forms. An email was sent to eligible participants confirming the study had closed at this time. Recruitment was restricted to this number due to the number of available photobooks.

As we conducted this research during the COVID-19 pandemic, one photobook was mailed to each participant at their home postal address. The photobook arrived in a cardboard box, which also contained a brief set of instructions (supplementary file 1), return postage, and a return postal address. Participants were not incentivised for taking part. The participants had up to 14 days to engage with the photobook in their own time prior to participation in a focus group.

### Data collection

After engaging in the photobook, the participants were invited to join a focus group session two weeks later, which was conducted online via Microsoft Teams. In total, four focus groups took place (*n* = 22), comprised of registered nurses (*n* = 12) and undergraduate nursing students (*n* = 10). All participants had experience in providing care to people living with dementia in a professional role, either as a registered nurse or a nursing student. Two participants withdrew from the study prior to data collection without providing reason. Focus group 1 (*n* = 6) and focus group 2 (*n* = 6) were comprised of registered nurses who were undertaking dementia-specific postgraduate modules. Focus group 3 (*n* = 4) and focus group 4 (*n* = 6) were comprised of undergraduate nursing students from adult, mental health, and learning disability fields of nursing. The focus groups lasted between 35–50 min in duration and took place between December 2020 and February 2021.

All focus groups were recorded, and consent was rechecked by the facilitator prior to commencement. Each focus group was facilitated by one member of the research team (GM) who was not involved in the development of the intervention, has clinical expertise in dementia, and has previous experience in the facilitation of focus groups. A semi-structured focus group guide, which was designed by the research team and reviewed by six people living with dementia, was used to guide discussion (supplementary file 2).

### Data analysis

Data were transcribed verbatim and subsequently analysed thematically using Braun and Clark’s six stage framework [50]. These stages included familiarisation with the transcripts, generation of initial codes, deciding on preliminary themes, reviewing the themes, defining the final themes, and writing up the findings. All four members of the research team participated in data analysis. AC led transcription, while all four authors participated in data familiarisation and generation of initial codes independently. Following a team meeting, all members of the research team decided on the preliminary themes with SD, GM, & GC reviewing and defining the final themes.

### Trustworthiness

Lincoln and Guba’s [51] recommendations on trustworthiness and qualitative research were followed throughout the research process. To ensure consistency and stability, one author (GM) completed all focus groups based on the same questions. Practicing reflexivity by implementing a reflexive journal to log the interviewer experiences alongside peer debriefing following the focus groups allowed recognition for any influence of data interpretation of own preconceptions. Detailed record keeping maintained throughout and a decision audit trail provides the rationales for decisions made and reflections on the data analysis. Participants were also provided a copy of their transcript (member checking).

## Results

Following thematic analysis of the data, three themes emerged: humanising the person behind the disease, constructing your own meaning about dementia, and innovative complimentary dementia education.

### Theme One: Humanising the person behind the disease

The most consistent theme to emerge from the data related to participant’s feelings that the photobook supported their personal understanding of humanising the person behind dementia. Participants explained that the combination of photographs and the person’s own personal writings offered a unique insight into the experience of dementia:

“We all hear about the symptoms and signs of dementia and the pathophysiology of it, but it’s hard to really understand what someone themselves is going through without having something like this…the pictures humanise the process of dementia”. Focus Group 3, Participant 1, Nursing Student.

The humanisation of dementia was something that resonated with most participants. During the focus groups, participants spoke of their role in caring for people in the advance stages of dementia. They believed that people living with advanced dementia often required more complex care due to the progression of their illness, for example assistance with eating and drinking due to dysphagia, assistance with elimination due to incontinence, and psychological care for symptoms of distress. Because of this, many participants focused more strongly on clinical or medical needs of the person. Following the introduction of the photobook, participants reflected on how the photobook could foster empathy for both new and experienced dementia care nurses.

“I think it kind of like just personalises dementia overall, like it’s not patient X with dementia. It’s this person, it’s his life, and he also has dementia. Like dementia doesn’t really define the person, which I think sometimes maybe carers can sometimes forget, mistakenly. So, I think it’s [the photobook] just a nice reminder: no matter how difficult [medically] this person is, this person could easily be your grandad”. Focus Group 4, Participant 5, Nursing Student.

The humanisation of dementia led to greater levels of self-perceived empathy amongst many participants. Both registered nurses and nursing students spoke about how the photobook gave them an opportunity to understand certain experiences that were common among people with dementia, for example disorientation, forgetfulness, and confusion. One aspect that particularly struck participants was the person’s insight into their illness as noted below:

“You weren’t told about this confusion; you’re actually seeing it through this writing.” Focus Group 3, Participant 1, Nursing Student.

“He does make these wee [written] notes … and he sort of refers to himself a wee bit as maybe having memory problems, but he doesn't see that as a big issue. So, it's maybe a journey that other people can see but you know for him it's just a thing [disease]. He's still a person”. Focus Group 1, Participant 2, Registered Nurse.

While the person living with dementia was a key focus for participants, many also commented on the importance of the family caregiver perspective which was present in the letter from his wife. Participants reflected on the importance of understanding the impact of dementia from the perspective of the family caregiver, and how this could often be neglected in their own nursing practice due to the high level of focus on the person with dementia. During the focus group interviews, participants collectively spoke about how powerful the letter was and felt that this even further humanised the person with dementia, by seeing the person through the eyes of a loved one.

“When you’re flicking through it [photobook], he's got quite a lot of like family pictures in it as well and it kind of hits you as well that, even though he has dementia, like how it’s affecting like the family circle around him…His wife said at the back [letter] things he used to do…and just how it affects others around you as well.” Focus Group 3, Participant 4, Nursing Student.

Overall, participants felt strongly that the photobook supported their professional and personal understanding of dementia. Many participants talked openly about how the photobook helped them to consider the person behind the dementia diseases in a way that they had not done before.

### Theme Two: Constructing your own meaning about dementia

All participants confirmed that the photobook was a unique way to learn about dementia. No participant had ever previously been involved with or come across an approach to dementia education like the photobook. Because participants had not encountered photographic arts like this before, they were often hesitant to trust their own interpretations. During focus groups, participants often qualified their observations, using phrases like *“maybe it’s just me reading too much into it,”* “*I was over thinking it,”* and *“I don't know if that’s relevant or not.”* Participants frequently described a feeling of confusion upon first encountering the photobook because it was very different to how they had previously learned. As participants progressed through the reading experience, however, they often gained confidence in their interpretations, and they gleaned new meaning from the material, as one participant stated:

“I think there was a lack of confidence when we first read it because you were trying to wrap your mind around the concept of what is actually happening in this book. But your confidence grows whenever you understand and re-read it, and you get more from it every time you read it, you get that something a little bit more from it”. Focus Group 3, Participant 1, Nursing Student.

It became apparent that nurses and nursing students felt more comfortable with traditional modes of learning, for example face-to-face lectures, small groupwork exercises, or teacher-led flipped learning. The participants attributed this comfort to the usual linear transmission of information from teacher to student, for example the pathophysiology of dementia or the importance of shared decision-making in palliative dementia care. Because the photobook was based on a constructivist pedagogy, it required all participants to construct their own meanings about dementia throughout their reading. Unlike more traditional modes of learning, there was not a clear ‘right’ or ‘wrong’ way to learn from the photobook.

“It's one of those things I think that you could read it, or you could just flick through it, and you could get nothing from it. But it actually challenges you to think … because some of the things I'm looking at, I'm thinking, that's my perception of that, but I don't know if that's the rationale as to why. That's just my interpretation of it … it seems very grey, there's no black and white with it, so it's really how much you want to take out of it”. Focus Group 1, Participant 4, Registered Nurse.

Another participant spoke about how the photobook helped them to construct their own meaning about dementia from it:

“The first glance just goes by random pictures … but then when you go through, when you get to understand what the person is trying to make us understand, or what the photographer wants to tell us, then you understand the real meaning. But first, you’re thrown off what is happening. Then the second time when you go through … yes, you understand what is happening and it’s related to that person with dementia”. Focus Group 2, Participant 5, Registered Nurse.

While all participants collectively described similar learning outcomes, in relation to humanising the person with dementia, the journey participants took in constructing their opinions was often unique. Some participants acknowledged that the non-chronological order of photographs, the abstract placement of some photographs (i.e., not in the centre of the page), and the lack of instruction (i.e., the photobook begins in the middle of a story with no firm introduction), were all akin to the dementia disease and symptoms of disorientation, distortion, and confusion. Participants, who all had prior experience of caring for someone living with dementia, were uniquely placed to understand these subtleties. The excerpts below highlight this discussion amongst participants.

“The way I like interpreted was like there was some of the photos were like split you know like there was something in the middle of them. So, the way I sort of seen it was like a gap in the memory”. Focus Group 4, Participant 1, Nursing Student.

“I quite liked that it wasn't consistent, like it wasn't just one page was pictures, one page was writing. It was just a bit all over the place, which I think kind of describes people with dementia. Like, there’s no one way of treating one person with dementia. Like, each person deals with their condition differently and so you’ve to kind of like … change your, like, approach to everyone differently, which I think is quite nice. It kind of brings up the fact that everyone just goes through this differently, I think.” Focus Group 4, Participant 3, Nursing Student.

As illustrated in the quotations, during the focus group participants enthusiastically shared their interpretations and learning from the photobook. As a result, participants asserted that some form of post-photobook debrief session was necessary to share collective learning experiences amongst peers. This was evidenced in the quote which follows:

“When I was reading this myself like I didn't really understand it but see coming on and talking to other people about it … it sort of gives you more of an insight.” Focus Group 3, Participant 2, Nursing Student.

Collectively, participants positively engaged with the photobook as an innovative style of learning. Despite initial uncertainty about how to use the photobook and what learning they had taken from it, focus group interviews illuminated positive experiences and meaningful learning. Participant recommendations regarding a formal debrief session are also likely to enrich learning further in the future.

### Theme Three: Innovative complimentary dementia education

The final theme that emerged from the data related to the future use of artist-produced photobooks in dementia care education. On-going dementia education is important for nurses and nursing students. There are a range of educational approaches to dementia education, however none of the study participants had experienced dementia education by using a photobook. Participants valued this innovative approach to dementia education, and they were often keen to explore ways in which the photobook could benefit other people. With consideration to the wider public, participants felt the photobook had the potential to be beneficial. However, they also believed that members of the public, or even some healthcare professionals with limited or no dementia experience, would be unlikely to construct their own meanings in the way that experienced practitioners had in this study, and perhaps would need additional support. This belief appeared to be validated by participants who had shared their copy of the photobook with a member of their family as noted here:

“From working with patients with dementia, I get why it is the way that it is. But from an outsider's point of view, [my husband] just thought it was a book full of photos and he just didn't understand why it was dementia friendly. But after explaining to him my perceptions of what was useful in it, then he got it. But it took the explaining for him to get it”. Focus Group 1, Participant 4, Registered Nurse.

In response to the varying experiences that readers would have about dementia, some participants suggested that the photobook should come with more instructions about how to use it and information about how the photobook could be interpreted. However, the idea of offering a reader more instruction is potentially at odds with the constructivist approach adopted by this intervention that intentionally leaves room for the reader to make their own meanings.

With regards to professional education, student nurses and nurses felt the photobook was an important and useful way to support learning about dementia. Participants felt that the photobook could complement existing dementia education that was already available, for example face-to-face teaching, groupwork, and e-learning. Specifically, participants believed that the photobook could support people working in healthcare in learning about person-centred dementia care, as noted in the excerpt below.

“I think in the nursing home this could be helpful for the new care assistants because we get a lot of girls who are 17 or 18 and they don't know a lot and many of them don't even understand dementia or what it means. And I think this could be useful for them to understand person-centred care”. Focus Group 1, Participant 5, Registered Nurse.

As an innovative approach to learning about how dementia effects a person, participants felt this resource should be shared with other healthcare professionals, informal carers, and the wider public.

## Discussion

Person-centred care has been long established as the cornerstone to good dementia care [52–54]. As a key basis of dementia care, it is important for healthcare workers to be able to empathise with people with dementia and understand symptoms of dementia, for example forgetfulness, confusion, or distress. Empathetic approaches to patient care are therefore grounded in personhood, which is said to be a status of equality and value between one human being and another, for example a nurse and a person with dementia [54]. Despite these recommendations, there are several threats to the personhood of individuals living with dementia, not caused by the disease but by societal treatment [53]. This devaluing and objectifying of others is referred to as malignant social psychology [10–56]. Examples of malignant social psychology include treating people with dementia like children (infantilisation), referring to the person using labelling language (labelling), not facilitating people with dementia to use the abilities they still have (disempowerment), or not acknowledging the reality of a person (validation) [52]. Research has shown that an important way to negate malignant social psychologies is through education which helps healthcare professionals understand the importance of the person in dementia care [57–61].

This study has highlighted that an artist-produced photobook about dementia has the potential to humanise people living with dementia by highlighting, through photography, how someone living with dementia retains their personhood throughout the advanced stages of their illness. As such, an artist-produced photobook has the potential to complement existing dementia care education by supporting participants to learn in an innovative way.

As a novel approach to dementia education, the artist-produced photobook facilitated participants to develop a greater understanding about dementia. The aim of this photobook was to facilitate nurses, and nursing students, to actively construct their own meaning of dementia from their previous experiences and beliefs. This underpinning constructivist pedagogy is already well established in higher education and is often associated with positive learning outcomes due to its ability to equip learners with authentic learning experiences which are directly relevant to individual context [62–65]. In this study, participants felt that professionals with expertise and knowledge about dementia were more likely to construct meaningful learning from the photobook than people with less knowledge. This feedback appears to validate the artist-produced photobook as an educational tool for a professional audience. Presently, most education about dementia is tailored to separate audiences of the public, informal caregivers, healthcare professionals, and dementia professionals [66–69].

Although the focus group was initially intended only as a means of data collection, the group discussion that took place in the focus groups proved to be vital to the process of learning from the photobook. Therefore, we recommend that arts-based educational interventions, such as this one, be accompanied by a facilitated group discussion. As educators in the field of medicine are, often, as unfamiliar with arts-based interventions and creative methods as nurses and nursing students, we have designed a discussion guide to support the implementation of *Thanks, Gd* in the classroom (supplementary file 3). This discussion guide is designed to foster a non-judgemental environment which supports participants to move beyond a scientific understanding of dementia diseases and toward a reflection about how they view and care for people with dementia [70]. To this end, art is uniquely placed. Greenslit, Price, and Malone have shown that exposure to artwork can enable viewers to “connect with a scientific idea or concept on a more emotional or aesthetic level or allow for a moment of critical review” [71]. Moreover, research on visual thinking strategies (VTS) in nursing education has indicated that group discussion about artwork can enhance nursing students’ skills of “collaboration and team working, fostering patient-centred care” [69]. These findings led participants to a shared conclusion which suggested that art-based interventions had the potential to complement existing approaches to dementia education at both pre-registration and post-registration level. Current UK policy guidance on dementia education affirms that dementia education must be underpinned by the lived experiences of people living with dementia [72–75]. Facilitation of arts-based educational interventions, such as *Thanks, Gd*, may have the potential to further promote awareness and understanding about the lived experience of dementia.

### Strengths and Limitations

The key strength of this study was that it explored an innovative approach to dementia education, an artist-produced photobook about dementia, from the perspective of both registered nurses and nursing students. To our knowledge, this is the first time an educational intervention of this type has been explored in nursing. While this study will make an important contribution to the evidence-base, the findings may be difficult to generalise in other parts of the world. The artist-produced photobook featured a man living with dementia in the United States of America, and this was viewed by participants who were living and practising in Northern Ireland. It is therefore difficult to estimate the impact this photobook would have in different countries and cultures. A further limitation relates to sampling and recruitment. This is because purposive sampling and self-nomination of participants was likely to mean that study participants were more enthusiastic about dementia than others. It is therefore unlikely that this sample truly reflects the views of the typical nurse or nursing student.

Another strength of this study is that the photobook was produced by a professional artist whose close family member was diagnosed with dementia. Visual artists are skilled at conveying meaning and tell complex stories through imagery. Therefore, using a photobook that has been designed and produced by a professional photographer, especially one with a personal relationship to dementia, is a strength of this study. Moreover, although the artist herself has not personally experienced the symptoms of dementia, she included her grandad’s written words that describe his feelings as his dementia progressed, thus providing an authentic insight into the lived experience of dementia.

## Conclusion

Using an artist-produced photobook as ABP in nursing education is an innovative way to learn about dementia. Nurses and nursing students articulated that this approach helped them to better understand the person behind the dementia diseases. As such, an artist-produced photobook is a useful complimentary resource for supporting professional education about dementia. Especially when paired with a facilitated group discussion, artwork such as this has the ability to foster personal reflection and enhance skills of collaboration. These activities contribute toward developing a practice of person-centred care among nurses and nursing students.

## Supplementary Information

**Additional file 1:** 

**Additional file 2:** 

**Additional file 3:** 

## Data Availability

The full dataset generated and analysed during the current study are not publicly available in order to maintain the privacy of the individuals interviewed during this study. De-identified data can be made available from the corresponding author on reasonable request.
