# Supplementary material for: Exploring nurse and nursing student experience of using an artist-produced photobook to learn about dementia
Source: BMC Nurs. 2022 Aug 25;21:237. doi: 10.1186/s12912-022-00991-2 (PMC9406272; doi:10.1186/s12912-022-00991-2)
Supplement: Supplementary file 1 — Additional file 1: [file 12912_2022_991_MOESM1_ESM.docx]

**Instruction Sheet**

**Title of Project:**

**Images and Empathy: Enhancing Positive Perceptions of Dementia among Nursing Students using an Artist-Produced Photobook**

**Upon receipt:**

- Retain this box so that you can reuse it when you return the book via post
- Take care not to lose the provided materials, including the return postage
- Do not remove the photobook from your house except to return it via post

**Using the materials:**

- Read the photobook in your own time and in your own way
- We estimate that you will need a minimum of 15 minutes in a quiet location with no interruptions to review the materials

**Returning the materials:**

- Reuse the box that the photobook arrived in and tape it securely
- Detach the address below and tape it securely on the box as a label

Contact Dr Gillian Carter or Savannah Dodd with any questions or concerns.

**Dr Gillian Carter | CONTACT INFORMATION REDACTED**

*Lecturer in Chronic Illness School of Nursing & Midwifery, Queen’s University Belfast*

**Savannah Dodd | CONTACT INFORMATION REDACTED**

*Photographer and PhD candidate in Anthropology, Queen’s University Belfast*

………………………………………………………………………………………………

Savannah Dodd

ADDRESS REDACTED

Belfast
