# Supplementary material for: Exploring nurse and nursing student experience of using an artist-produced photobook to learn about dementia
Source: BMC Nurs. 2022 Aug 25;21:237. doi: 10.1186/s12912-022-00991-2 (PMC9406272; doi:10.1186/s12912-022-00991-2)
Supplement: Supplementary file 2 — Additional file 2: [file 12912_2022_991_MOESM2_ESM.doc]

**Focus-Group Interview Guide**

**Title of Project:**

**Images and Empathy: Enhancing Positive Perceptions of Dementia among Nursing Students using an Artist-Produced Photobook**

**[All focus-groups to be moderated by at least two members of the research team]**

The purpose of this focus-group is to discuss your experience of viewing the photobook *Thanks, Gd.* There are a few areas I would like to discuss about your experience. If any participant wishes to stop or withdraw from the interview at any time, please remain silent and turn off your camera. A member of the research team will follow up with you after to confirm your withdrawal from the study.

***Topic Areas:***

**Practicalities of the photobook**

- How long did you spend with the photobook?
- Where did you view the photobook?
- Did you share it with anyone?
- Did you have any challenges viewing the photobook?
- What did you like most about the photobook?
- Did you enjoy the experience of viewing the photobook?
- Would you recommend the photobook to others in your field?

**Learning from the photobook**

- What did you learn or gain from viewing this photobook?
- What do you think others could learn from this photobook?
- Should this photobook be used in healthcare education?

**Impacts of the photobook**

- Did this photobook influence or change how you feel about people with dementia?
- Has this photobook influenced or changed the way that you approach your work with people with dementia?
- Who do you think could benefit from viewing this photobook?

**Limitations of the photobook**

- Have you viewed a photobook before?
- Have you engaged with photographic art about dementia before?
- What did you dislike about this photobook?
- How might this photobook be improved?

Thank you for your time, this has been very helpful and I would be extremely interested in any other thoughts or feelings you have and would like to share to help me better understand your experience or is there anything you would like me to go back to?
