# Supplementary material for: Exploring nurse and nursing student experience of using an artist-produced photobook to learn about dementia
Source: BMC Nurs. 2022 Aug 25;21:237. doi: 10.1186/s12912-022-00991-2 (PMC9406272; doi:10.1186/s12912-022-00991-2)
Supplement: Supplementary file 3 — Additional file 3: [file 12912_2022_991_MOESM3_ESM.doc]

**Discussion Guide for *Thanks, Gd***

1. What is this photobook about? How do you know that?
2. What was your favourite part of the photobook? Why?
   1. Which was your favourite photograph? Why?
3. Was there anything you struggled with when reading the photobook?
   1. Did anyone else have the same struggle?
   2. Did anyone view it differently?
4. Why do you think the artist made this photobook?
5. What came to mind for you as you were reading the photobook?
   1. Did you find this representation of dementia relatable from your experience?
   2. Did anything in the photobook surprise you? Why or why not?
6. How did you feel after reading the photobook?
   1. Did you feel differently at the end than you did at the start?
7. What is your biggest take-away from the photobook?
   1. Do you feel like this photobook impacted you in any way?
   2. Do you think you will remember it in a year?
